# Colonic mucosal biopsy location can not affect the results of mucosal metabolomics and mucosal microbiota analysis in IBS

**DOI:** 10.3389/fmed.2023.1183484

**Published:** 2023-06-07

**Authors:** Huiting Zhu, Yanli Zhang, Shiyu Du, Huifen Wang, Yue Zheng

**Affiliations:** ^1^Department of Internal Medicine, Hebei Medical University, Shijiazhuang, China; ^2^Department of Gastroenterology, First Hospital of Qinhuangdao, Qinhuangdao, China; ^3^Department of Gastroenterology, China-Japan Friendship Hospital, Beijing, China

**Keywords:** irritable bowel syndrome, ileocecal mucosa, sigmoid, metabolomics, microbiota, lipid

## Abstract

**Objective:**

To compare and analyze the mucosal metabolites and mucosal microbiota of different parts of colon in patients with IBS.

**Methods:**

A total of 10 patients with IBS-D and six healthy controls (HC) were enrolled. All enrolled participants underwent two biopsies of the ileocecal and sigmoid colon during colonoscopy. Metabolomic profiling of one piece of tissue was conducted using desorption electrospray ionization-mass spectrometry (DESI-MS), and the gut flora of the other piece was examined using 16S rRNA sequencing. The metabolic profiles and flora of the ileocecal and sigmoid colonic mucosa in each group were further analyzed in this study.

**Results:**

(1) Principal components analysis (PCA) indicated that mucosal metabolites did not differ in different parts of the colon in either the IBS-D or HC groups. (2) In the mucosal microbiome analyses, no differences between the microbiota of the two parts of the colon were found by using Principal Co-ordinates Analysis (PCoA). In IBS group, comparing with sigmoid mucosa, the chao1 richness indice was higher and the Shannon index was lower in the ileocecal mucosa (*p* = 0.40, *p* = 0.22). However, in the HC group, microbiome analysis of the ileocecal mucosa showed lower values for Chao 1 and Shannon indices than those of the sigmoid colon mucosa (*p* = 0.06, *p* = 0.86). (3) Compared with the HC group, 1,113 metabolic signal peaks were upregulated, whereas 594 metabolites were downregulated in the IBS-D samples. Moreover, the PCA of the metabolites showed significant separation between the IBS-D and HC groups. (4) Chao1 expression was significantly higher in the mucosal microbiota with IBS-D than in the HC (*p* = 0.03). The Shannon index was lower in IBS-D, but the difference was not statistically significant (*p* = 0.53). PCoA revealed a significant difference in the microflora structure between the IBS-D and HC groups.

**Conclusion:**

The mucosal metabolic profile and mucosal flora structure of the colon were similar, despite different locations in IBS and healthy subjects. IBS had abnormal colonic mucosal metabolism and flora disturbances.

## 1. Introduction

Irritable bowel syndrome (IBS) is a common functional gastrointestinal disorder, with a prevalence of 11% (1). IBS imposes a great burden on both economics and psychology for patients and their families, and consumes medical resources for healthcare systems and society (2, 3). Exploring the specific pathogenesis of IBS remains a goal that researchers are continuously pursuing.

Recent metabonomics techniques examine small molecules in biological samples and detect subtle changes reflecting different physiological and pathological conditions, which have also been used to identify potential metabolic profiles for IBS. Recently, several metabonomics technologies, such as hydrogen nuclear magnetic resonance (H-NMR), liquid chromatography mass spectrometry (LC–MS), gas chromatography mass spectrometry (GC–MS), and high pressure liquid chromatography–tandem mass spectrometry (HPLC–MS), have revealed abnormal glucose, lipid, and amino acid metabolism in patients with IBS (4–6). These results were mostly obtained from fecal, blood and urine samples. Mucosal barrier dysfunction and low-grade mucosal inflammation have been suggested to be involved in IBS pathogenesis, and identifying colon mucosal metabolites could help us understand IBS pathogenesis (7–10). Some experiments have found that there may be some differences in the cellular structure of different parts of IBS colon (11, 12). Will these diferences affect the results of IBS mucosal metabolomics analysis?However, reports on the metabolomics of the colon mucosal tissues are rare. There are no relevant reports on whether different colon sampling sites affect the results of mucosal metabolomics. As a new metabolomics technology, desorption electrospray ionization-mass spectrometry imaging (DESI-MSI) can simultaneously obtain sample metabolite information and present the spatial distribution of metabolites. However, it has not yet been applied to the study of intestinal mucosal metabolism in IBS.

Gut microflora dysbiosis is a major mechanism underlying IBS (13–15). The collection of feces is often used as a sampling method for intestinal flora research because of its simplicity. However, some studies have reported differences in fecal microbiota and mucosal flora (16–18). The distribution of Firmicutes and Actinobacteria increased in feces, whereas that of Bacteroidetes and Proteobacteria decreased in stools compared to the mucosal flora (15). Experiments demonstrated differences in the rectal mucosal flora with fecal microbiota, even though it was the colon closest to the stools (16). Theoretically, mucosal flora can restore information about the flora in the internal environment to the greatest extent possible. The current findings on the colonic mucosal microbiota in patients with IBS from different studies lack consistency. Some investigators found abnormal colonic mucosal flora of Actinobacteria, Proteobacteria and Bacteroides in IBS patients, while others found that the mucosal microbiota in IBS patients was not significantly different from that in the general population (19, 20). After analyzing and comparing these research methods, we found that the biopsy sites of colonic mucosa were different in different experiments. There are no relevant reports on whether different positions of the colonic mucosal biopsy affect the results of studies on the mucosal flora.

The study applied DESI-MSI and 16S rRNA sequencing for ileocecal and sigmoid colon mucosa in IBS-D patients and healthy controls. This study aimed to compare the differences in mucosal metabolites and microbiota from different parts of the colon and to explore the characteristics of mucosal metabolism and microbiota in IBS-D.

## 2. Materials and methods

### 2.1. Research objects and experimental design

We performed a prospective study in IBS-D patients and healthy controls (HC). Ten adult patients with IBS-D who met the Rome IV criteria were recruited from the Department of Gastroenterology at our hospital between May 2022 and August 2022. The severity of IBS-D was measured using the IBS Symptom Severity Scale (IBS-SSS), which includes five items (severity and frequency of abdominal pain, bowel habit dissatisfaction, abdominal distension, and life interference). Six healthy volunteers from the community participated in this study. Healthy subjects without organic intestinal disease or gastrointestinal symptoms were recruited as controls.

The inclusion criteria for all enrolled subjects were as follows: age between 18 and 65 years; normal blood count; alanine aminotransferase (ALT), alkaline phosphatase (ALP), and serum creatinine within reference values; and normal thyroid function. Exclusion criteria included the following: history of polyps in 3 years; malignant tumors; history of major gastrointestinal surgery; diarrhea due to other medical illnesses (e.g., inflammatory bowel disease, hyperthyroidism, diabetes mellitus, neurological diseases); chronic opioid or antidepressant use; pregnancy; and breastfeeding. None of the participants were allowed to take any prebiotics, probiotics, antibiotics or antidiarrheal medications within 4 weeks before recruitment.

Colonoscopies were performed in both patients with IBS-D and healthy controls. All enrolled participants underwent two biopsies of both the ileocecal and sigmoid colons during colonoscopy. Two biopsy specimens from each part of the colon were used for DESI-MSI and 16S rRNA sequencing. The study protocol was approved by the Human Ethical Committee of the China-Japan Friendship Hospital, and all participants provided informed consent.

### 2.2. Sample preparation

The ileocecal and sigmoid specimens were snap-frozen in liquid nitrogen immediately after biopsy removal and stored at −80°C until further processing. One piece of the frozen samples from the same location was embedded in 5% sodium carboxymethyl cellulose (CMC). Frozen sections (20 μm thick) were stored at −80°C for DESI–MSI and 9 μm thickness for stained with hematoxylin and eosin (H&E). The other piece was examined using 16S rRNA sequencing for intestinal flora analysis.

### 2.3. DESI-MSI

All MSI experiments were performed using a SYNAPT G2-Si HDMS DESI XS instrument (Waters, Milford, MA, United States) and a Harvard Apparatus Pump11 Elite. Glass slides containing 20 μm slices were subjected to DESI–MS imaging in the positive and negative ion modes over the mass range m/z 500–1,100 and 100–1,000. Then 1,000 peaks with the highest intensities were selected. The spray solvent for DESI was methanol/water in a ratio of 98:2, containing 200 ppb leucine encephalin, and injected at a rate of 3 μL/min. The parameter settings were as follows: capillary temperature, 150°C; nitrogen spray, 0.45 MPa; capillary voltage, 3.5 kV. Tissues were performed in constant velocity scan mode with a velocity of 100 μm/s and set at a spatial resolution of 50 μm to acquire DESI–MS images.

Four equal-area regions of interest (ROI) were selected on the mucosal layer of each DESI image and compared with the H&E staining images. EZinfo 3.0 (Waters) was used to further process the DESI-MS imaging data. Multivariate analyses were applied to the metabolite data. A principal component analysis (PCA) model served was used to identify potential differences between the two groups. Volcano scatter plots were used to identify differentiating metabolites, for which the fold change for each metabolite between IBS-D and healthy controls was calculated (i.e., metabolite A in the IBS group/metabolite A in the healthy group). Significance [−log10 (*p* < 0.05), Student’s *t*-test] versus log2 (mean fold change) was plotted. The mass spectrometry peaks were annotated to known compounds based on the HMDB^1^ and LIPID MAPS databases.

### 2.4. Detection of intestinal flora

Another frozen intestinal mucosa sample from the same location was used for high-throughput sequencing of intestinal flora. Total genome DNA from samples was extracted using CTAB/SDS method. By using the universal primers 341F (CCTAYGGGRBGCASCAG) and 806R (GGACTACNNGGG- TATCTAAT), the V3-4 regions of the bacterial 16S rRNA gene were amplified. Individual amplification products were purified and a sequencing library was constructed. Library quality was assessed on a Qubit@2.0 Fluorometer (Thermo Scientific) and Agilent Bioanalyzer 2,100 system, the library was sequenced on an Illumina NovaSeq platform, and 250 bp paired-end reads were generated, spliced, and filtered to obtain clean data. Operational taxonomic units (OTUs) clustering and species classification were performed. Visualization and comparison of intestinal flora were performed using a Principal Co-ordinates Analysis (PCoA) model with a permutation testing algorithm to detect microbiota variations between the two groups.

### 2.5. Statistical analysis

Statistical analyses were performed using the dedicated statistical software SPSS (version 26.0; IBM Corp, NY, United States) and GraphPad Prism version 8.0. Student’s *t*-tests were used for comparisons between the IBS-D and HC groups. Statistical significance was defined as *p* < 0.05.

## 3. Results

In total, our study comprised 10 patients with IBS-D (Average age = 34.20 ± 6.81 years, BMI 24.83±4.31kg/m^2^) and six healthy subjects (Average age = 38.33 ± 10.62 years, BMI 24.34±2.23kg/m^2^). There were no significant differences in age or BMI between the patients and healthy subjects. The mean IBS-SSS in patients with IBS-D was 257.7. A total of 32 biopsy specimens were obtained from the 16 subjects. Specifically, 12 biopsies from healthy controls (six from the ileocecal colon and six from the sigmoid colon) and 20 from patients with IBS-D (10 from the ileocecal colon and 10 from the sigmoid colon) were collected.

### 3.1. Metabolomic profiling in different parts of colon mucosa

**Figure 1:**
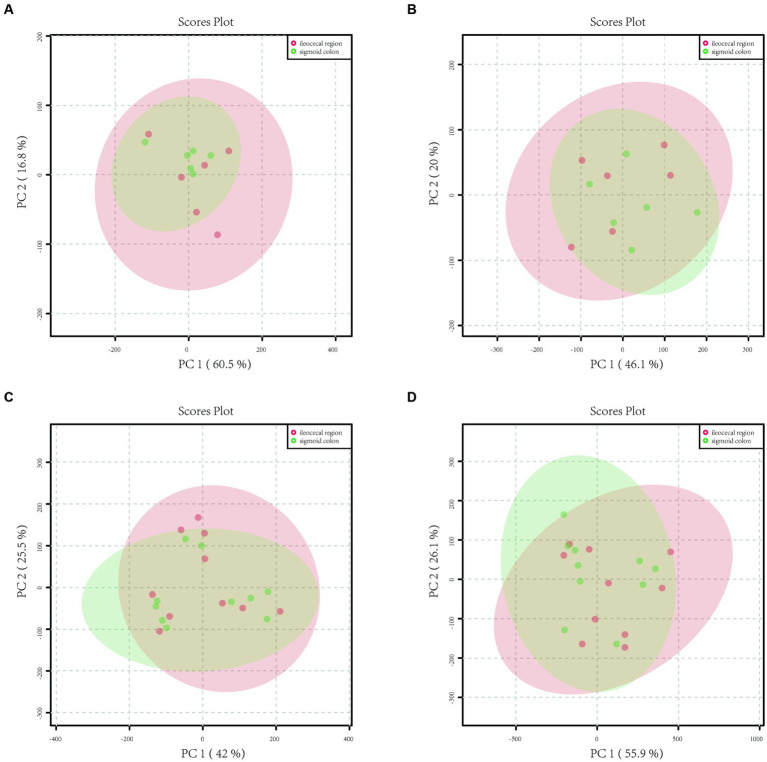
Principal component analysis (PCA) model plots of the metabolite profiles of HC and IBS in both positive and negative ion modes. **(A)** The PCA score scatter plot in positive ion mode showed that the metabolite profiles of healthy controls of the sigmoid colon (green) and ileocecal region (red) samples no differed. **(B)** The PCA score scatter plot in negative ion mode showed that the metabolite profiles of healthy controls of the sigmoid colon (green) and ileocecal region (red) samples no differed. **(C)** The PCA score scatter plot in positive ion mode showed that the metabolite profiles of IBS-D patients of the sigmoid colon (green) and ileocecal region (red) samples no differed. **(D)** The PCA score scatter plot in negative ion mode showed that the metabolite profiles of IBS-D patients of the sigmoid colon (green) and ileocecal region (red) samples no differed.

In the mucosa of the ileocecal and sigmoid colon in the HC group, 2,712 metabolite peaks were detected in the positive ion mode, and 2,655 metabolite peaks were identified in the negative ion mode. PCA revealed that the mucosal metabolites were indistinguishable between the two parts of the colon (Figures 1A,B) in both positive and negative ion modes.

A total of 4,846 metabolite peaks were detected in the ileocecal and sigmoid mucosa of patients with IBS-D in the positive ion mode and 4,837 metabolite peaks in the negative ion mode. The PCA models are displayed in Figures 1C,D, respectively. Compared with the sigmoid mucosa, the data points for metabolites in the ileocecal mucosa overlapped, showing an unseparated state in the charts. In both the positive and negative ion modes, the metabolic profiles of the two parts of the colon did not differ in the PCA model (Figures 1C,D).

### 3.2. Mucosal microbiota composition and distribution in different parts of colon

**Figure 2:**
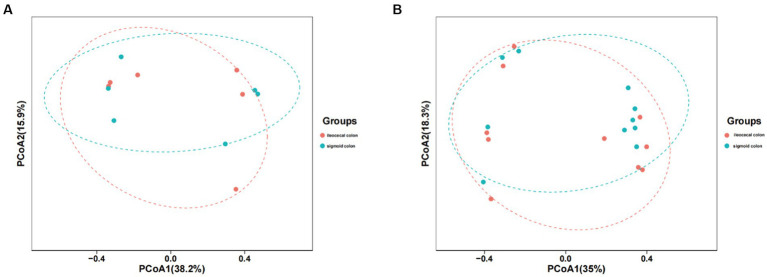
PCoA model plots of the mucosal microbiome of healthy subjects and IBS-D patients. **(A)** The PCoA of microbiota for two parts of colon in HC group. **(B)** The PCA of microbiota for two parts of colon in IBS-D group.

The mucosal microbiota had 952,225 raw sequences, with an average of 79,352 per sample, in the HC group. The Chao1 and Shannon indices in the ileocecal colon were lower than those in the sigmoid colon; however, the difference was not statistically significant (*p* = 0.06, *p* = 0.86, respectively) (Table 1). PCoA comparisons of the mucosal microbiota between the two parts of the colon could not distinguishable (Figure 2A).

**Table 1:** The Chao1 and Shannon index of healthy controls and IBS-D patients.

		Ileocecal colon	Sigmoid colon	*p*
HC	Chao1	690.97 ± 71.18	860.20 ± 181.52	0.06
	Shannon	4.24 ± 1.96	4.40 ± 0.88	0.86
IBS-D	Chao1	811.66 ± 122.83	754.28 ± 163.05	0.40
	Shannon	3.65 ± 1.29	4.53 ± 1.71	0.22

*p* < 0.05 statistically significant difference.

In patients with IBS-D, 1587187 raw read sequences were detected with an average of 79,359 per sample. Chao1 expression in the ileocecal colon was higher than that in the sigmoid colon, whereas the Shannon index of the ileocecal colon was lower, with no statistically significant difference between the two groups (*p* = 0.40, *p* = 0.22) (Table 1). PCoA revealed that the mucosal microbiota was not distinguishable between the two parts of the colon in IBS-D (Figure 2B).

### 3.3. Comparison of the metabolites profile and mucosa flora between IBS-D and HC

#### 3.3.1. Metabolites profile of mucosa

**Figure 3:**
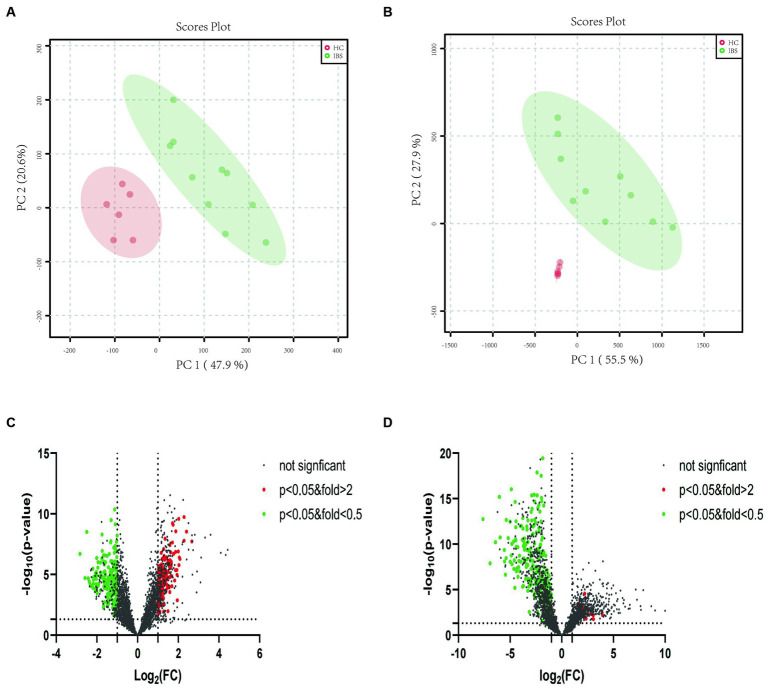
PCA plots and volcano plots of the metabolite profiles of IBS-D and HC in both positive and negative ion modes. **(A)** The PCA score scatter plot in positive ion mode showed that the metabolite profiles of colonic mucosa of the IBS-D (green) and HC (red) samples differed obviously. **(B)** The PCA score scatter plot in negative ion mode showed that the metabolite profiles of colonic mucosa of the IBS-D (green) and HC (red) samples differed obviously. **(C)** The volcano plots of the differential metabolites in positive ion mode. **(D)** The volcano plots of the differential metabolites in negative ion mode. Every spot represents a metabolite in colon mucosa. The red spots stand for the metabolites which were markedly higher detected in IBS-D than in HC, while the green spots stand for these significantly lower detected values in IBS-D. The black spots mean that there is no significantly different metabolites between the two groups.

In the ileocecal colon mucosa of both IBS-D and HC, 8916 metabolite peaks were detected, and 8,566 metabolite peaks were detected in the sigmoid colon mucosa. Metabolites of the ileocecal colon mucosa were subjected to PCA. PCA score charts are shown in Figure 3A (positive ion mode) and Figure 3B (negative ion mode). There was no overlap of data points in the intestinal mucosa of patients with IBS-D compared to those of the HC group, and the data were clearly classified in different areas. The obvious separation status in the score plots suggests that the mucosal metabolites in the two groups were well distinguished. This indicates that there is an obvious difference in mucosal metabolites between patients with IBS-D and healthy controls. PCA could distinguish mucosal metabolites between patients with IBS-D and healthy controls in both positive and negative ion modes (Figures 3A,B). The volcano plots (Figures 3C,D) showed the compounds that met both FC > 2 and *p* < 0.05 between the two groups in both the positive and negative ion modes. When compared with the HC group, the higher detected metabolites of the colon mucosa in patients with IBS-D are represented by red spots, lower ones are represented by green spots, and black spots indicate no significant differences between the two groups. Compared to the HC group, we found that 401 metabolite peaks of IBS-D were upregulated and 304 peaks of IBS-D were downregulated in the positive ion mode (Figure 3C), whereas 712 peaks were upregulated and 290 peaks were downregulated in the negative ion mode (Figure 3D).

Moreover, we focused on lipid metabolite analysis. The metabolite class categories were determined using the Human Metabolome Database (HMDB) and Lipid Map Classification. A total of 159 upregulated lipid species in IBS-D were detected within six lipid categories (10 fatty acids, 22 glycerolipids, 66 glycerophospholipids, 7 sphingolipids, 23 sterols and 31 prenol lipids). A total of 475 downregulated lipids from seven lipid categories were observed: 78 fatty acids, 111 glycerolipids, 153 glycerophospholipids, 10 sphingolipids, 63 sterols, 58 prenol lipids, and 2 N-acyl amino acids (Figures 3C,D).

#### 3.3.2. Mucosal microbiota composition and distribution

**Figure 4:**
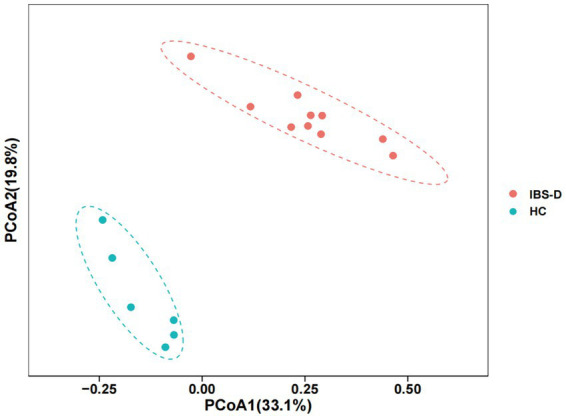
The PCoA of microbiota in the colon mucosa of IBS-D group and HC group. PCoA1 represents the first principal co-ordinate, PCoA2 represents the second principal co-ordinate, and percentage represents the corresponding contribution ratio. Different dots and colors in the figure represent samples and groups, respectively.

In both the IBS-D patients and HC groups, the mucosal microbiota had a total of 1,204,299 raw sequences, with an average of 75269 per sample. The Chao1 index in the IBS-D group was significantly higher than that in the HC group (*p* = 0.03). The Shannon index for IBS-D was slightly lower than that for HC, but the difference was not statistically significant (Table 2). Based on the PCoA, there was an overall difference in the mucosal bacterial composition between patients with IBS-D and HC (Figure 4).

**Table 2:** The Chao1 and Shannon index comparing IBS-D group and HC group.

	IBS-D	HC	*p*
Chao1	811.66 ± 122.83	690.97 ± 71.18	0.03*
Shannon	3.65 ± 1.29	4.24 ± 1.96	0.53

* *p* < 0.05 statistically significant difference.

**Figure 5:**
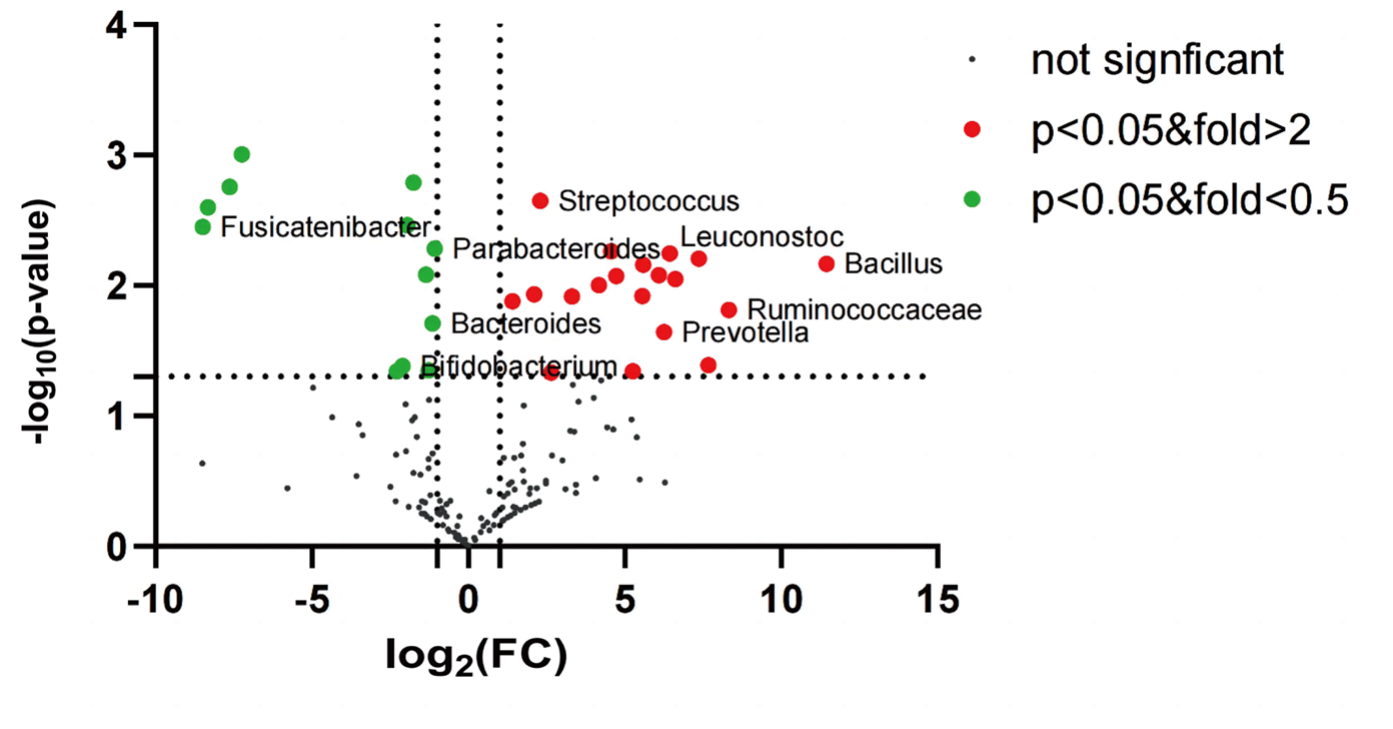
The volcano plot of mucosal microbiota for IBS-D and healthy control (genus level). Red dots: the abundance of microbiota in IBS-D group was up-regulated compared with that in HC group. Green dots: the abundance of microbiota in IBS-D group was down-regulated compared with that in HC group. Black dots: there was no significant difference between IBS-D group and HC group, and *p* value <0.05 was statistically significant.

Further comparison of the species differences at the genus level of the intestinal mucosa flora revealed that the differences between the IBS-D and HC groups were significant. Of these, Bacillus, Ruminococcaceae, Prevotella, Oscillibacter, Clostridium, Acinetobacter, Leuconostoc, Klebsiella, Cronobacter, Fastidiosipila, Methylobacterium, Lactococcus, Candidate_division_TM7_norank, Pseudomonas, Enterococcus, Rothia, Streptococcus, Raoultella and Lachnospiraceae_uncultured were significantly upregulated in the IBS-D group compared to those in the HC group. However, Parabacteroides, Bacteroides, Bifidobacterium, Faecalibacterium, Blautia, Halanaerobium, Chromohalobacter, Enterobacter, Coprococcus, Ruminiclostridium, LachnospiraceaeUCG-004 and Fusicatenibacter were downregulated in the IBS-D (Figure 5).

## 4. Discussion

In this study, we examined the mucosal metabolites and mucosal microbiota of the ileocecal and sigmoid colon in patients with IBS-D and healthy controls. We detected that the mucosal metabolites and structure of the mucosal microbiota from different parts of the colon were consistent, not only in patients with IBS-D but also in healthy controls. Moreover, the mucosal metabolic profile and mucosal flora structure of patients with IBS-D were differ from those of healthy controls.

Based on qualitative and quantitative analyses, the metabolism of the intestinal mucosa could be used to evaluate the functions of the human body systems and their relative changes, which follow physiological or pathological stimuli. DESI-MSI, a new metabonomics technology, can be used to analyze gut mucosal metabolites and perform *in situ* imaging. Metabolites in the colonic mucosa can regulate mucosal permeability, increase cellular calcium influx, promote neuropeptide release, induce inflammatory pain and aggravate smooth muscle contraction (8, 9). In previous studies, colon mucosa was collected from the ileocecal region, sigmoid colon and rectum (21). There are no relevant reports on whether different colon sampling sites affect the results of mucosal metabolomics. In this study, similar mucosal metabolites were obtained from different parts of the colon. These similarities existed not only in patients with IBS-D but also in healthy controls. In addition to having the same source of embryonic and structural tissues, it may also be related to the integrity of the colonic mucosa. When the colonic mucosa is broken or inflamed, the metabolites must change (22, 23). Studies comparing the inflamed colonic mucosa in patients with Crohn’s disease (CD) with healthy subjects found that glycosaminoglycans (GAGs) increased considerably in CD (24). In addition, the inflamed colonic mucosa in CD showed a significant decrease in C18:2n6 and C18: 3n3 levels compared to the non-inflamed mucosa in CD (25). Fatty acid metabolism may be abnormal in the mucosa of colon cancer patients compared to the surrounding normal tissues (26). It is well known that the colonic mucosa is intact in both IBS-D patients and healthy people. Thus, we believe that the mucosal metabolic profiles of different parts of the colon were similar in an individual when the mucosa was intact.

At the same time, we also found that there was a metabolic disorder in colonic mucosa of patients with IBS-D, such as 1,113 metabolites were upregulated and 594 metabolites were downregulated. Previous studies have shown that alterations in metabolite production may be associated with the symptoms of IBS. Stool samples from patients with IBS-D were analyzed using an untargeted LC–MS approach, and the results showed that both tryptophan and tryptamine were significantly increased (21). Solakivi et al. (27) found that the levels of some long-chain polyunsaturated fatty acids in the serum of patients with IBS decreased, whereas those of some monounsaturated fatty acids increased. It was also found that the levels of short-chain fatty acids, such as propionic acid and butyric acid, in the feces and serum of patients with IBS were higher than those in healthy controls (27). This study found that patients with IBS-D had lipid metabolism abnormalities similar to those in previous studies; however, we found more species of lipid metabolism abnormalities, such as fatty acids (FAs), glycerolipids, glycerophospholipids (GLP), sphingolipids (SM), prenol lipids (PR), steroids and steroid derivatives. This may be because DESI-MSI is more sensitive to lipid detection.

Ninety-five percent of the body’s microbiota reside in the gut, primarily in the colon. Are there any differences in the species and quantity of microflora in the mucosa of different parts of the colon in an individual? In our study, the abundance and diversity of the ileocecal mucosal flora in healthy individuals were slightly lower than those of the sigmoid colon. However, in patients with IBS-D, the abundance of ileocecal mucosal flora was slightly higher and the diversity was slightly lower than that of the sigmoid colon. However, the all differences were not statistically significant. Therefore, we speculated that the structure of the mucosal flora in different parts of the colon was consistent in both healthy and IBS-D patients and recommend biopsy of the mucosa of any part of the colon as a research sample for the study of colonic flora.

We also found that patients with IBS-D had colonic dysbacteriosis. Patients with IBS-D showed higher colonic microbiota abundance and slightly lower diversity than healthy controls. At the genus level, Prevotella, Streptococcus, Ruminococcaceae, Klebsiella, Rothia and Raoultella increased in the colonic mucosa of patients with IBS-D, while Bifidobacterium, Bacteroides and Fusicatenibacter decreased. Our results were similar to those of other experimental studies that showed that Prevotella, Lactobacillus and Bifidobacterium were imbalanced in the colons of patients with IBS-D. Prevotella can activate Th17 cells through Toll-like receptor 2, followed by the release of IL-23 and IL-1 from Th17 cells and mediate mucosal inflammation (28). Bifidobacteria not only produce short-chain fatty acids to increase intestinal permeability, but can also modulate the immune system by producing tolerogenic DCs (29–31). Beneficial bacterial reduction and harmful bacterial growth could be involved in the pathogenesis of IBS by damaging the mucosal barrier, decreasing pain thresholds and activating the mucosal immune response.

The relationship between microbiota and metabolites is fantastic. Microbiotas in the colon can affect the physiological activities of themselves and their hosts through the metabolites produced by their metabolism. Collinsella can produce butyrate in intestine, and butyric acid-producing bacteria in previous studies could alleviate inflammatory bowel disease, obesity and type 2 diabetes (32, 33). In IBS-D patients, Bacillus could secret acetylcholine, which might potentially lead to diarrhea and abdominal pain (34). At the same time, microbiota can also affect the growth and reproduction of other gut flora through metabolites, and then regulate the life activities of the host. Bifidobacteria produce short chain fatty acids via fermentation of carbohydrates, and these metabolities can turn the luminal environment acidic inhibiting adherence of invasive bacteria (35).

However, this study had limitations such as small sample size, semi-quantitative and non-targeted DESI-MSI method, and lack of correlation analysis between metabolites and flora. Because a large number of metabolites were included in the studies, caution is necessary when interpreting the results. However, it is highly encouraging that patients with IBS-D and healthy controls were fairly well differentiated.

## 5. Conclusion

In summary, this study found that the mucosal metabolic profiles and mucosal flora structure of the ileocecal and sigmoid colons were similar in both healthy individuals and patients with IBS-D. The poor consistency of IBS colonic mucosal flora is not caused by mucosal location. It is recommended that single-site biopsy of the colon be used for mucosal metabolomics and microbiome studies. This study also found that patients with IBS-D have abnormal colonic mucosal metabolism and mucosal flora disturbances. However, whether there is a correlation between metabolic disorders and flora disturbances and their roles in the pathogenesis of IBS should be further explored.

## Data availability statement

The original contributions presented in the study are included in the article/supplementary material, further inquiries can be directed to the corresponding author.

## Ethics statement

The studies involving human participants were reviewed and approved by the Human Ethical Committee of China-Japan Friendship Hospital (Beijing, China). The patients/participants provided their written informed consent to participate in this study.

## Author contributions

HZ and YZ were performed the material preparation, data collection, analysis, and wrote the first draft of the manuscript. All authors commented on previous versions of the manuscript, contributed to the study conception, design, read, and approved the final manuscript.

## Conflict of interest

The authors declare that the research was conducted in the absence of any commercial or financial relationships that could be construed as a potential conflict of interest.

## Publisher’s note

All claims expressed in this article are solely those of the authors and do not necessarily represent those of their affiliated organizations, or those of the publisher, the editors and the reviewers. Any product that may be evaluated in this article, or claim that may be made by its manufacturer, is not guaranteed or endorsed by the publisher.

## Footnotes

^1^http://www.hmdb.ca
